# Light-induced tumor theranostics based on chemical-exfoliated borophene

**DOI:** 10.1038/s41377-022-00980-9

**Published:** 2022-11-11

**Authors:** Zhongjian Xie, Yanhong Duo, Taojian Fan, Yao Zhu, Shuai Feng, Chuanbo Li, Honglian Guo, Yanqi Ge, Shakeel Ahmed, Weichun Huang, Huiling Liu, Ling Qi, Rui Guo, Defa Li, Paras N. Prasad, Han Zhang

**Affiliations:** 1 Institute of Pediatrics, Shenzhen Children’s Hospital, Shenzhen, Guangdong China; 2 Shenzhen Engineering Laboratory of phosphorene and Optoelectronics; International Collaborative Laboratory of 2D Materials for Optoelectronics Science and Technology of Ministry of Education, Shenzhen Institute of Translational Medicine, Department of Otolaryngology, Shenzhen Second People’s Hospital, the First Affiliated Hospital, Institute of Microscale Optoelectronics, Shenzhen University, 518060 Shenzhen, China; 3 Department of Microbiology, Tumor and Cell Biology (MTC), Karolinska Institute, Stockholm, Sweden; 4 Shenzhen Medical Ultrasound Engineering Center, Department of Ultrasonography, Shenzhen People’s Hospital, Second Clinical Medical College of Jinan University, First Clinical Medical College of Southern University of Science and Technology, 518020 Shenzhen, China; 5 Optoelectronics Research Center, School of Science, Minzu University of China, 100081 Beijing, PR China; 6 Nantong Key Lab of Intelligent and New Energy Materials, College of Chemistry and Chemical Engineering, Nantong University, 226019 Nantong, Jiangsu China; 7 Key Laboratory of Biomaterials of Guangdong Higher Education Institutes, Guangdong Provincial Engineering and Technological Research Centre for Drug Carrier Development, Department of Biomedical Engineering, Jinan University, 510632 Guangzhou, China; 8 Department of Core Medical Laboratory, the Sixth Affiliated Hospital of Guangzhou Medical University, Qingyuan People’s Hospital, Qingyuan, Guang Dong Province China; 9 Department of Laboratory Medicine, Shenzhen Children’s Hospital, Shenzhen, Guangdong China; 10 Institute for Lasers, Photonics, and Biophotonics and Department of Chemistry, University at Buffalo, State University of New York, Buffalo, NY USA

**Keywords:** Biophotonics, Nanoparticles

## Abstract

Among 2D materials (Xenes) which are at the forefront of research activities, borophene, is an exciting new entry due to its uniquely varied optical, electronic, and chemical properties in many polymorphic forms with widely varying band gaps including the lightest 2D metallic phase. In this paper, we used a simple selective chemical etching to prepare borophene with a strong near IR light-induced photothermal effect. The photothermal efficiency is similar to plasmonic Au nanoparticles, with the added benefit of borophene being degradable due to electron deficiency of boron. We introduce this selective chemical etching process to obtain ultrathin and large borophene nanosheets (thickness of ~4 nm and lateral size up to ~600 nm) from the precursor of AlB_2_. We also report first-time observation of a selective Acid etching behavior showing HCl etching of Al to form a residual B lattice, while HF selectively etches B to yield an Al lattice. We demonstrate that through surface modification with polydopamine (PDA), a biocompatible smart delivery nanoplatform of B@PDA can respond to a tumor environment, exhibiting an enhanced cellular uptake efficiency. We demonstrate that borophene can be more suitable for safe photothermal theranostic of thick tumor using deep penetrating near IR light compared to gold nanoparticles which are not degradable, thus posing long-term toxicity concerns. With about 40 kinds of borides, we hope that our work will open door to more discoveries of this top-down selective etching approach for generating borophene structures with rich unexplored thermal, electronic, and optical properties for many other technological applications.

## Abstract (web summary)

Chemical-exfoliated borophene and its application in multi-imaging guided photothermal therapy.

## Graphical abstract

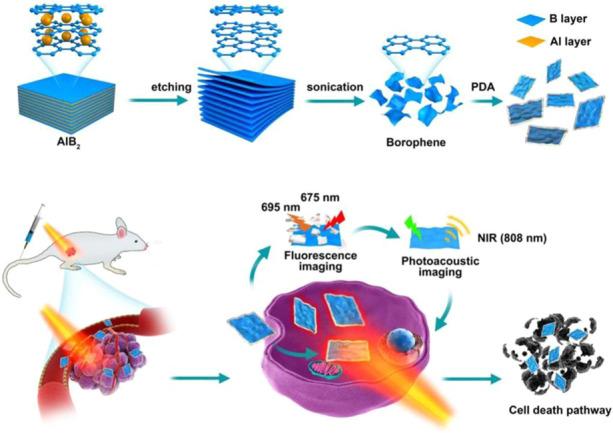

## Introduction

Light has made profound impacts on clinical practice for diagnosis and treatment. The modern use of light in medicine went through a rapid improvement in the understanding of fundamental light–matter interactions since the 19th century. When absorbed by a biological tissue or an added exogenous probe, the resulting emission shows the microstructure and ingredient of the tissue, serving for optical imaging and diagnostics. On the other hand, the photoirradiation of the tissue through introduced photosensitizer can also be used in therapy via photothermal effect, photochemical reactions (photodynamic effect), or another biological process (optogenetic).

Recent advances in biomedical optics have enabled integrated photonics technologies with nanotechnology and biomaterials. The two-dimensional (2D) nanomaterials, inspired by the great success of graphene^1^, have undergone tremendous expansion in recent years to include MoS_2_^2–5^, MXenes^6–9^, black phosphorus (BP)^10–21^, bismuthene^22–24^, antimonene^25–28^, tellurene^29–35^, selenene^36,37^, BP-analog materials^38–41^, 2D metal^42,43^, MOF^44,45^, etc. Recently, borophene, as the neighbor of graphene, has aroused great attention because it is the lightest 2D material up to now and is revealing many unique properties different from graphene^46,47^. With polymorphism and anisotropic structure^48–50^, borophene contributes to numerous unique characteristics such as mechanical flexibility^51,52^, metallicity^53^, optical transparency^54^, etc. Metallicity is the most unique attribute of borophene in comparison with semimetals or semiconductor, and thus it is promising for optically transparent electrode^54^. On the contrary, the semiconducting property can also be found in the γ-B28 phase monolayer and a strong photoluminescence signal is observed at ~626 nm corresponding to its direct band gap of 2.25 eV^55^. Moreover, the combined result of low mass and small bending stiffness is high phonon velocity, leading to efficient thermal transport^56^.

As is well-known, owing to a covalent bonding framework in bulk boron, borophene fabrication remains a great challenge. Theoretically, a triangular lattice with periodic holes, grown on metal substrates^49,57^, is predicted to be more stable^58,59^. This striped-phase borophene was first demonstrated experimentally through molecular beam epitaxy (MBE) growth on Ag(111) by Mannix et al.^53^ under ultrahigh-vacuum conditions. Moreover, the chemical vapor deposition (CVD) was used to grow atomically thin borophene on Cu foils^55^. In addition to these bottom-up growth, a top-down strategy was employed by Ji et al.^60^ to prepare borophene through synergetic thermal oxidation and liquid-phase exfoliation. Sonochemical technique was also used to exfoliate bulk boron by Ranjan et al.^61^. The rare bottom-up fabrication investigations illustrate the difficulty in fabrication of borophene from non-layer bulk B and thus limit any scope of wide application.

**Fig. 1:**
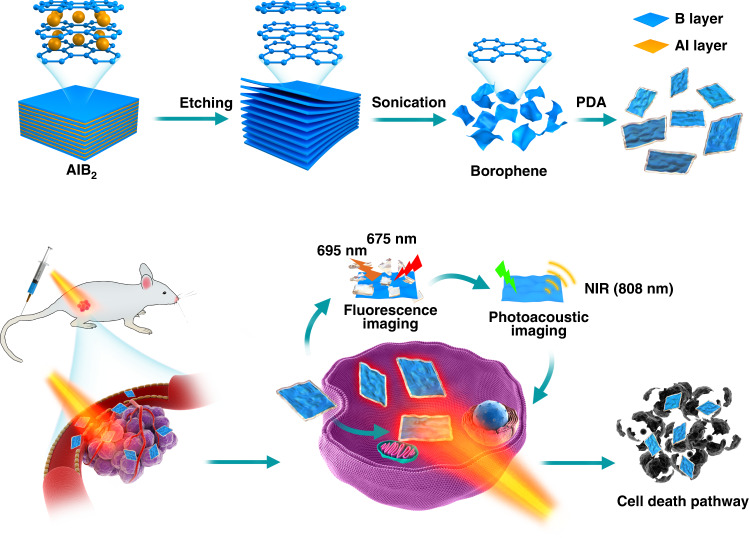
Schematic illustration. Similar to the fabrication process of MXene, the borophene was obtained through etching its precursor. Then, the modified borophene was applied in multi-imaging guided photothermal therapy

Herein, we demonstrate the light-induced tumor theranostics of the newly-fabricated borophene, as shown in Fig. 1. The borophene was produced by employing acid selective etching of the AlB_2_ precursor. A surprising finding is that while HCl etching produces borophene by dissolving Al, etching by HF yields Al sheets by dissolving B. Our unique method can overcome the hurdle in direct exfoliation from the covalently-bonding bulk boron. Similar to the exfoliation of MXene^62–64^, ultrathin and large borophene nanosheets can be obtained with a high yield. The relatively mild etching environment maintains the integrity of the borophene structure. This as-prepared borophene has significant absorption at 800 nm in the near IR region to produce the significant photothermal effect which can be used for photoacoustic imaging and photothermal cancer therapy. Using surface modification with polydopamine (PDA), we developed a biocompatible smart delivery nanoplatform of B@PDA which exhibits low toxicity, enhanced cellular uptake, strong photoacoustic signal at 800 nm, and photothermal therapy.

## Results

### Fabrications

**Fig. 2:**
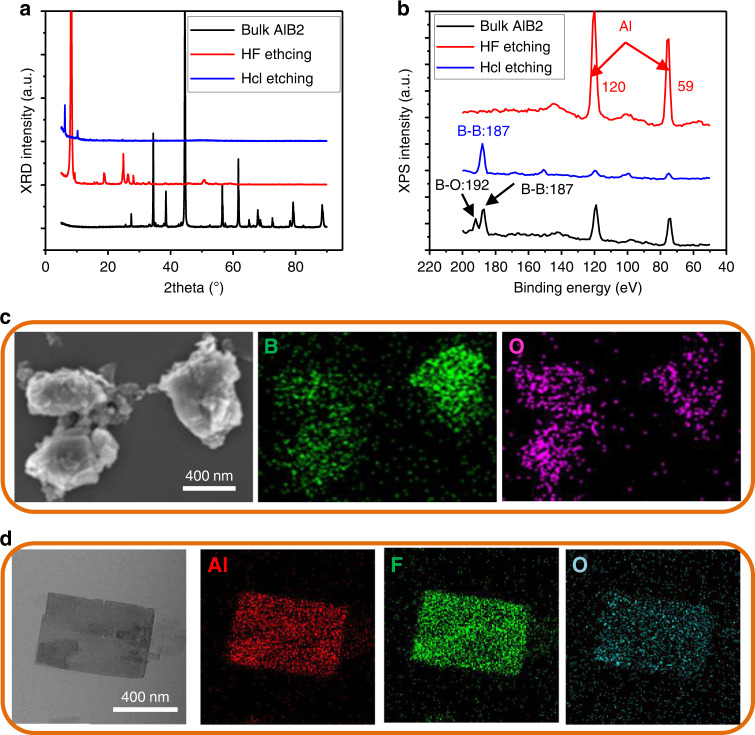
Chemical etching techniques for borophene. **a**, **b** XRD and XPS patterns of bulk AlB_2_, boron product after HCl etching and aluminum product after HF etching. **c**, **d** EDS mapping after HCl and HF etching, respectively

For the etching of borophene, suitable etching agent needs to be chosen. The etching efficacy of HCl and HF were compared. It was surprising to find that the boron ingredient can be obtained and aluminum can be etched out using the HCl etching process, while it is the opposite situation for the HF etching process (Fig. 2). As shown in Fig. 2a, the samples for HCl etching and HF etching show different X-ray diffraction (XRD) peaks from the precursor of AlB_2_. For the HCl etched sample, the most intense XRD peaks of AlB_2_ vanish and a peak at a small 2θ of 6^o^ appears, showing the ultrathin dimension of the B product exfoliated from bulk AlB_2_. The sample after HF etching shows peaks similar to the XRD pattern to AlF_3_^65^ and Al_2_O_3_^66^, with a strong XRD peak at 2θ of ~8^o^ indicative of ultrathin nanosheets. The chemical composition for both etching products was characterized by X-ray photoelectron spectroscopy (XPS) (Fig. 2b)^67,68^. After HCl etching, it is observed that the main component is the boron element^68^, while the HF etched product revealed Al, O and F elements, illustrated by the observed typical XPS patterns (Figs. 2b and S1)^69^. The energy-dispersive X-ray spectroscopy (EDS) mapping in the scanning transmission electron microscopy (STEM) mode shows the B, O without Al elements after HCl etching (Fig. 2c) and the Al, F, and O elements without B after HF etching (Fig. 2d). The quantitative analysis of the elemental composition is further performed by EDS analysis (Fig. S2). It is clearly observed that the B element accounts for 95.88% of weight after HCl etching, while HF etching can completely remove the B element, leaving Al, O and F elements. These systematic characterizations collectively prove that acid selective etching is an efficient strategy for achieving a boron product from its precursor of AlB_2_ and HCl is a suitable etching agent.

### Characterizations

**Fig. 3:**
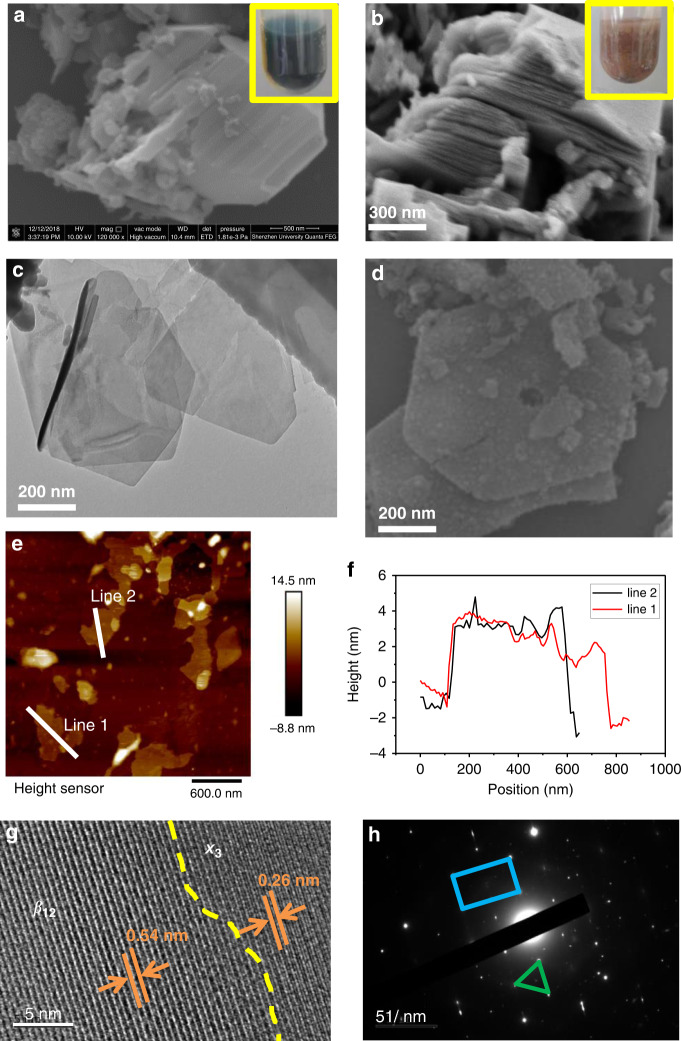
Characterizations of the newly fabricated borophene. **a** Scanning electron microscope (SEM) image of bulk AlB_2_. Inset shows the black color of bulk AlB_2_ before etching. **b** Book page-like morphology after HCl etching. Inset shows the red color of etching products from AlB_2_. **c**, **d** Transmission electron microscope (TEM) and SEM image of the borophene nanosheets. **e**, **f** The ultrathin thickness of borophene nanosheets characterized by atomic force microscope (AFM). **g** Crystal lattice characterized by the high-resolution TEM. **h** Selected-area electron diffraction (SAED) of borophene. The blue rectangle and green triangle correspond to the β_12_ structure and χ_3_ structure, respectively

After confirming a successful chemical etching process, morphological characterizations of the etched products are further conducted (Fig. 3). Figure 3a shows the SEM image of bulk AlB_2_. After HCl etching, a book page-like structure can be observed, showing successful extraction of the Al layer from AlB_2_ (Fig. 3b). The insets in Fig. 3a, b show the stark color contrast before and after etching. Further, with the sonication process, large and ultrathin borophene nanosheets with several hundreds of nm can be exfoliated (Fig. 3c, d). In particular, the nanosheets appear to be hexagonal, which may be due to the honeycomb structure in bulk AlB_2_^70^. The thickness of the borophene nanosheets is measured to be ~4 nm (Fig. 3e, f), exhibiting the high aspect ratio of lateral size and thickness.

Through the high-resolution TEM image (left part, Fig. 3g), the interatomic distance can be observed to be 0.54 nm, which agrees with the β_12_ model of borophene^67^. For the right part, the distance of 0.26 nm indicates the occurrence of also the χ_3_ phase. The diffraction pattern (Fig. 3h) further proves the coexistence of both the β_12_ and χ_3_ phases. Such an intermediate phase may be formed because of the absence of a supporting substrate, which is similar to the case of sonochemical exfoliated borophene^61^. Therefore, the final borophene phase depends only on the energetics of the lattice. The β_12_ phase possesses an out-of-plane structure with minimum energy. This metastable structure of the mixed-phase might have higher energy than that of β_12_, maintained by the special etching solution environment.

**Fig. 4:**
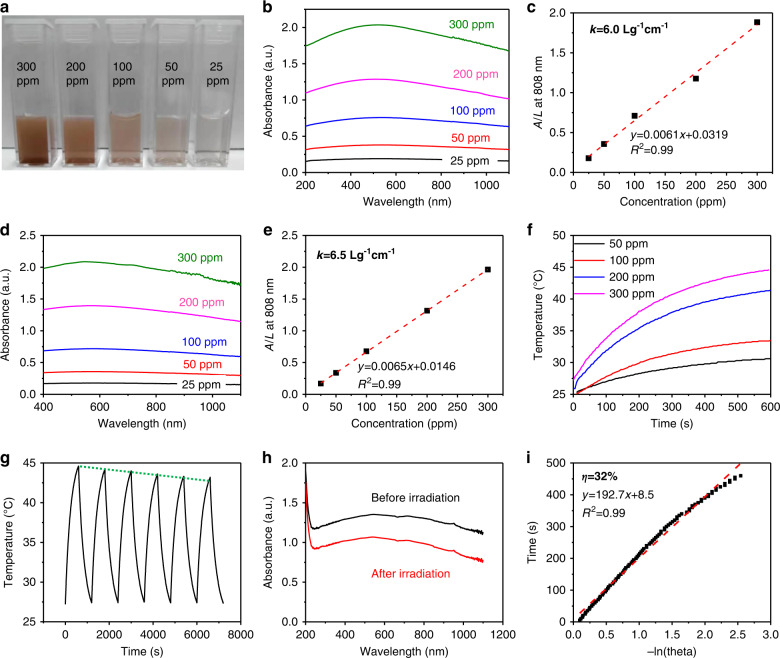
Photothermal characterizations of borophene. **a**–**c** Photograph, absorbance, and extinction coefficient of naked borophene. **d**, **e** Absorbance and extinction coefficient of B@PDA. **f** Photothermal temperature increases at different concentrations of B@PDA. **g** Periodic photothermal cycles. **h** Stability is shown by the absorbance before and after irradiation. **i** The calculation for photothermal conversion efficiency

Furthermore, the photothermal performance of the as-prepared borophene is characterized^13,43,71,72^. For the naked borophene, the dispersions for different concentrations are shown in Fig. 4a and the corresponding absorbance is shown in Fig. 4b. The extinction coefficient (k) is determined to be 6.0 Lg^−1^cm^−1^ (Fig. 4c), comparable to that of Ge nanosheets (5.962 Lg^−1^cm^−1^)^73^ and higher than those of gold nanorods (3.9 Lg^−1^cm^−1^)^74^ and antimonene (3.6 Lg^−1^cm^−1^)^75^, as shown in Table S1. To further enhance the absorption and biocompatibility, polydopamine (PDA) is chosen to decorate the borophene^76–78^. For PDA decorated borophene, the extinction coefficient can be enhanced to be 6.5 Lg^−1^cm^−1^ at 808 nm (Fig. 4d, e). In photothermal study, the temperature of B@PDA increases much faster than in pure PDA as laser irradiation time increases (Figs. 4f and S6). A gradually decaying photothermal performance of B@PDA for six cycles can be observed (Fig. 4g) indicating gradual degradation of the nanostructure. The absorbance decrease after laser irradiation also proved the degradability of B@PDA (Fig. 4h). The photothermal efficiency is calculated to be 32% (Fig. 4i), which is higher than MoS_2_^79^ and Ti_3_C_2_^80^ nanosheets (Table S2). The photothermal performance of the as-prepared materials at different pH values were also investigated (Fig. S3). The results show that acidic conditions slightly affect the photothermal properties of B@PDA, which may be related to accelerated degradation. Notably, the B@PDA possess photothermal performance upon NIR II laser (1064 nm) irradiation, but weakly compared to 808 nm irradiation (Figs. S4 and S5).

### In vitro anticancer efficacy assay of B@PDA

**Fig. 5:**
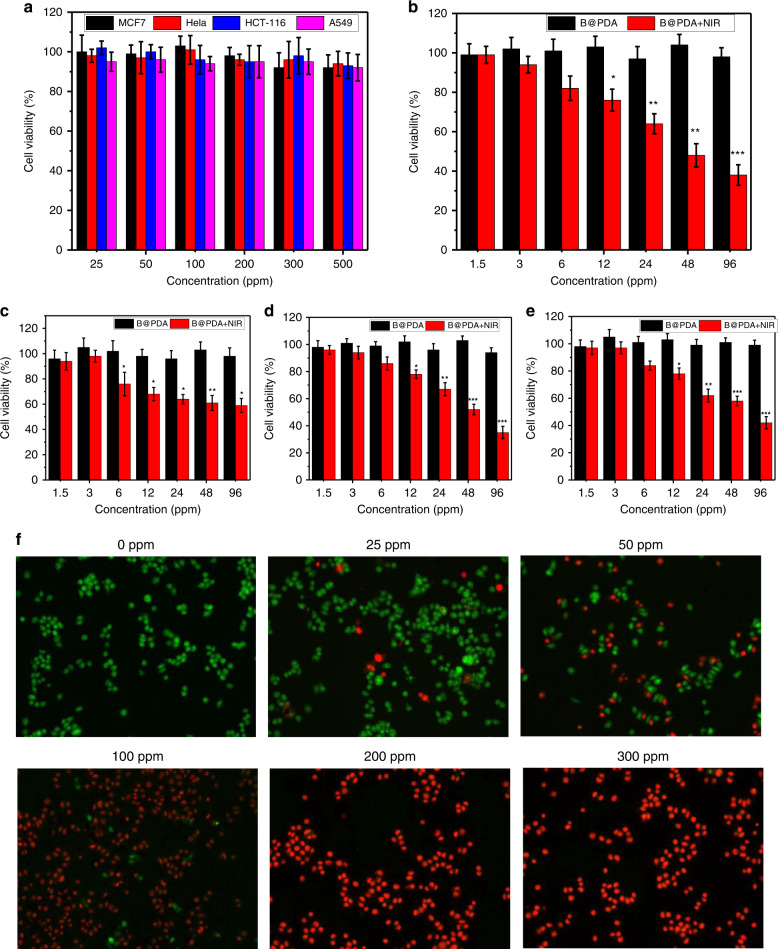
Cytotoxicity and photothermal killing experiments of B@PDA. **a** Relative viability of HCT-116, HeLa, A549, and MCF7 cells after exposure in B@PDA-based medium (25–500 ppm) for 48 h. **b** Relative viability of HCT-116 cell incubated with B@PDA (1.5, 3, 6, 12, 24, 48, and 96 ppm) for 4 h and after photothermal treatment (1 W cm^−2^, 808 nm, 10 min,). **c**–**e** were treated with the same condition as (**b**) for HeLa, MCF7, and A549, respectively. **f** Corresponding laser scanning confocal microscopy (scale bars, 50 µm for all panels) of HCT-116 cells stained with PI (dead cells, red) and calcein AM (live cells, green)

Good biocompatibility of B@PDA is the first requisite for its biomedical application. Therefore, in vitro cytotoxicity of B@PDA was tested in several different tumor cells, including HCT-116, HeLa, A549 and MCF7. The CCK-8 assay was used to measure the cells’ viability. These cells were incubated with B@BPA at different concentrations of 25–500 ppm for 48 h and no obvious cytotoxicity was observed for the tested cells even at the high concentration of 500 ppm (Fig. 5a). This result proves the low cytotoxicity of B@PDA and its potential for biomedicine in the first step, which was also verified on noncancerous cells (Figs. S10 and S11). After this confirmation, the photothermal killing effect of the B@PDA was further checked. The cells were firstly cultured with B@PDA for 4 h, and then were irradiated using an 808 nm laser (1 W cm^−2^, 10 min). The CCK-8 assays were used to measure the cell viability after another 24 h’s incubation. Figure 5b demonstrates a dose-dependent photothermal killing effect in HCT-116 cells. Figure 5c–e show similar results on other cells. In contrast, pure PDA exhibited negligible cell photothermal killing effect, probably because most of the PDA was lost during washing (Fig. S9). A laser scanning confocal microscopy (LSCM) assay in HCT-116 cancer cells provides similar results through staining cells after laser irradiation. The red fluorescence represents dead cells from propidium iodide (PI) and the green fluorescence represents live cells from Calcein-AM (Fig. 5f), which shows the obvious photothermal killing effect of the B@PDA.

### Endocytosis

**Fig. 6:**
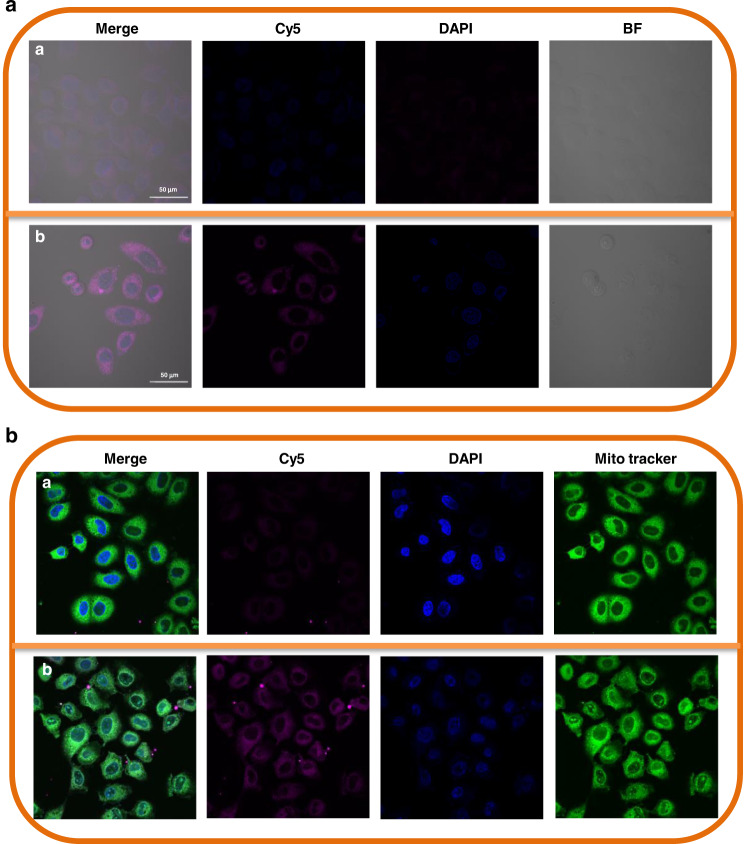
The pH-sensitivity and distribution in lysosome and mitochondria of Cy5-B@PDA. **a** CLSM images of HCT-116 cells cultured with Cy5-B@PDA at different pH environments after 3 h (Scale bar: 20 μm): (a) pH 7.4; (b) pH 6.5. **b** The localization of Cy5-B@PDA in mitochondria when HCT-116 cells were incubated with Cy5-B@PDA at 37 °C: (a) 30 min; (b) 3 h (Scale bar: 50 µm)

PDA with pH-sensitive property is promising for weakly acidic tumor microenvironment delivery^76–78^. To show the photothermal killing mechanism of B@PDA, the intracellular tracking of B@PDA was made through a coating of Cy5. The uptake ability of Cy5-B@PDA by HCT-116 cells in different pH environments (pH 7.4 and 6.5) was observed by LSCM. After treatment with Cy5-B@PDA for 15 min, the intracellular fluorescent intensities of Cy5-B@PDA at pH 6.5 and 7.4 environments were similar. Interestingly, the fluorescence of Cy5-B@PDA at pH 6.5 (Fig. 6a(a)) was stronger than that at pH 7.4 after 3 h (Fig. 6a(b)). This result demonstrates that PDA can facilitate cellular penetration of B@PDA in the acid tumor environment.

When HCT-116 was incubated with Cy5-B@PDA for 30 min, red Cy5 fluorescence was observed in the mitochondria (Fig. 6b(a)). After 3 h’s incubation of Cy5-B@PDA with HCT-116 cells, the red Cy5 fluorescence increased significantly (Fig. 6b(b)). The above results demonstrated that Cy5-B@PDA could target mitochondria, and further facilitate the following reactive oxygen species (ROS)-based therapy.

**Fig. 7:**
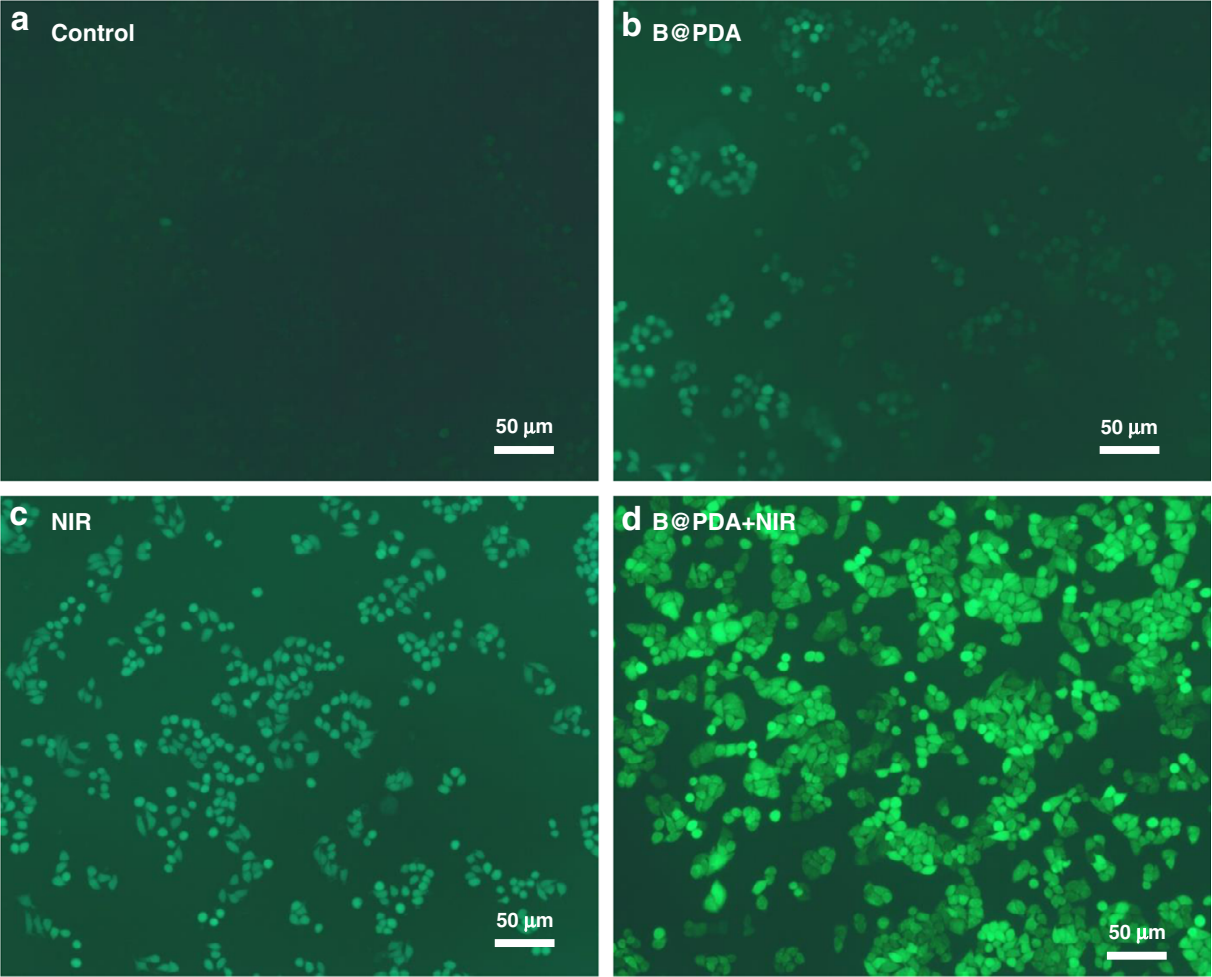
ROS measurement. **a** Control. **b** The HCT-116 cells were treated with B@PDA. **c** The HCT-116 cells were exposed to 808 nm irradiation (10 min, 1 W cm^−2^). **d** HCT-116 cells were cultured with B@PDA, and the cells were then irradiated with 808 nm (10 min, 1 W cm^−2^)

The ROS are highly reactive species produced from O_2_ metabolism^81,82^. The toxicity mechanism of some inorganic nanomaterials is related to ROS^83^. The level of ROS induced by B@PDA treatment of HCT-116 cells without laser irradiation was higher than the control (Fig. 7a, b). In the NIR laser irradiated cells, the ROS value increased considerably compared with the control (Fig. 7c). In the B@PDA + NIR group, the induced ROS quantity is the maximum (Fig. 7d). Therefore, the B@PDA-based tumor cell killing can be attributed to the production of ROS.

### In vivo imaging

**Fig. 8:**
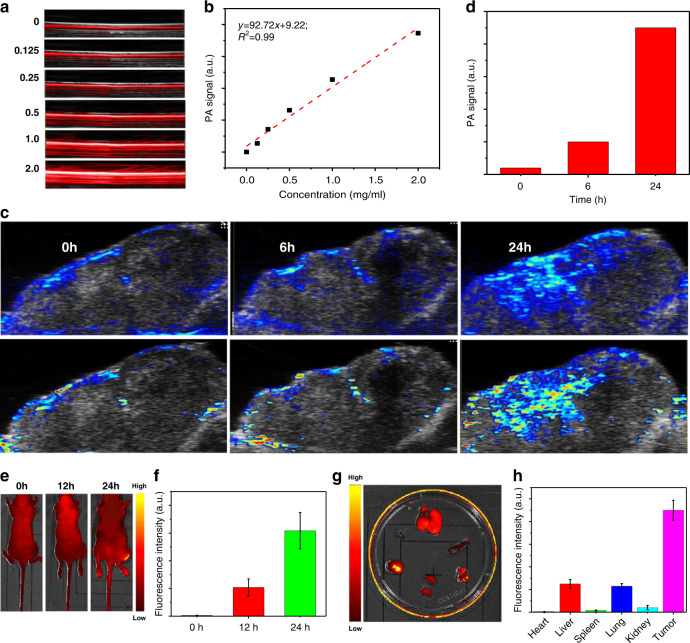
Multi-modal in vivo imaging and bio-distribution of B@PDA. **a** In vitro PA images of B@PDA vs. concentration (0, 0.125, 0.25, 0.5, 1, and 2 µg µl^−1^). **b** PA images of B@PDA vs. concentration (0, 0.125, 0.25, 0.5, 1, and 2 µg µl^−1^). **c** PA aqasximages in tumor site at time points of 1, 6, and 24 h post-injection. **d** Quantitative calculation of PA signal in PA images (**c**). **e** In vivo fluorescence images in tumor site of Cy5.5-labeled B@PDA during 24 h. **f** Quantitative calculation of Cy5.5-labeled B@PDA locating in the tumor site. **g** Fluorescence images of major organs at 24 h post-injection. **h** Quantitative biodistribution of B@PDA in BALB/c mice

It was revealed that nanomaterials induce endothelial leakiness (NanoEL) and exhibit enhanced permeability and retention (EPR) effect, leading to enhanced tumor-targeting ability^84–86^. The in vivo theranostic efficacy of the B@PDA-based platform was further explored. Recently, photoacoustic (PA) imaging has shown its potential in photothermal-based acoustic imaging technique in deep tumor^87–89^. Since the as-prepared B@PDA exhibits excellent photothermal performance, the B@PDA was further tested for PA imaging. Figure 8a shows a dose-dependent PA signal. A linear relation of the PA signal with the concentration of B@PDA was observed in Fig. 8b. In contrast, the photoacoustic signal of pure PDA is significantly weaker than that of B@PDA (Figs. S7 and S8). For the in vivo test, mice bearing HCT-116 tumor were injected with B@PDA systemically through caudal vein injection. After injection, the PA signal was measured at time points of 0, 6, and 24 h. A strong PA signal was observed 24 h post-injection of B@PDA at the tumor site (Fig. 8c). Moreover, a quantitative calculation of the PA signal further proved the strongest signal intensity at 24 h (Fig. 8d). These characterizations showed that B@PDA can be employed as a good PA contrast agent for guiding tumor therapy.

Furthermore, fluorescence imaging of animals was also conducted. The fluorescence signal in tumor sites at time points of 0, 12, and 24 h was measured using an in vivo imaging system. The very strong fluorescent signal appears post 24 h caudal vein injection of Cy5.5-labeled B@PDA at the tumor site (Fig. 8e, f). Then, the mice were killed and the organs were collected for fluorescence imaging. The tumor tissue shows the strongest fluorescent signal (Fig. 8g). Quantitative biodistribution of Cy5.5-labeled B@PDA in BALB/c mice was measured (Fig. 8h), which further confirmed the tumor-targeting ability of B@PDA. Therefore, a multi-modal imaging platform based on B@PDA can be achieved.

### Toxicity

**Fig. 9:**
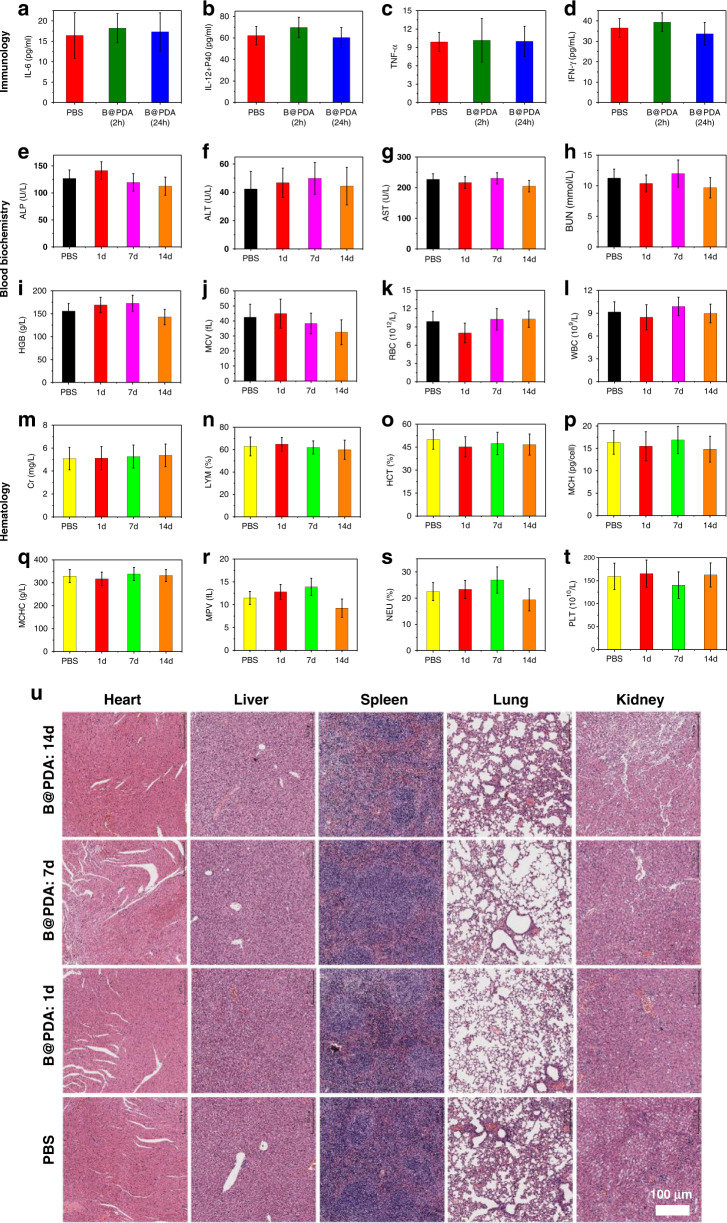
In vivo toxicity test. All the parameters are for healthy BALB/c mice injected with B@PDA and PBS. **a**–**d** Serum levels of immunology index. **e**–**t** Blood hematology and biochemistry data were collected after intravenous injection. **u** H&E staining of major organs

Before in vivo treatments, the toxicity of B@PDA was evaluated through in vivo test. The mice were injected with B@PDA and PBS intravenously (i.v.), respectively. For immune analysis, serum samples were gathered at 2 h and 24 h after injection. The IL-6, IL-12+P40, TNF-α, and IFN-γ levels in the B@PDA group were almost the same as those of the PBS group (Fig. 9a–d), demonstrating that B@PDA cannot cause any evident cytokine response. In the histology and hematology assay, the healthy mice were i.v. injected with B@PDA and PBS. The blood was gathered at 1, 7, and 14 days post-injection. The serum biochemical index was further measured, including urea nitrogen (BUN), alkaline phosphatase (ALP), aspartate aminotransferase (AST), and alanine aminotransferase (ALT) (Fig. 9e–h). For blood routine analysis, red blood cells (RBC), hemoglobin (HGB), white blood cells (WBC), mean corpuscular volume (MCV), mean corpuscular hemoglobin concentration (MCHC), mean corpuscular hemoglobin (MCH), platelet (PLT), neutrophil (NEU), hematocrit (HCT), creatinine (Cr), mean platelet volume (MPV), and lymphocyte (LYM) counts were detected (Fig. 9i–t). No significant statistical difference in all the parameters was observed between B@PDA and PBS-treated groups. These results show that B@PDA does not induce any obvious inflammation in BALB/c mice. Moreover, the H&E staining shows no distinct tissue damage sign in major organs (Fig. 9u).

### In vivo photothermal therapy

**Fig. 10:**
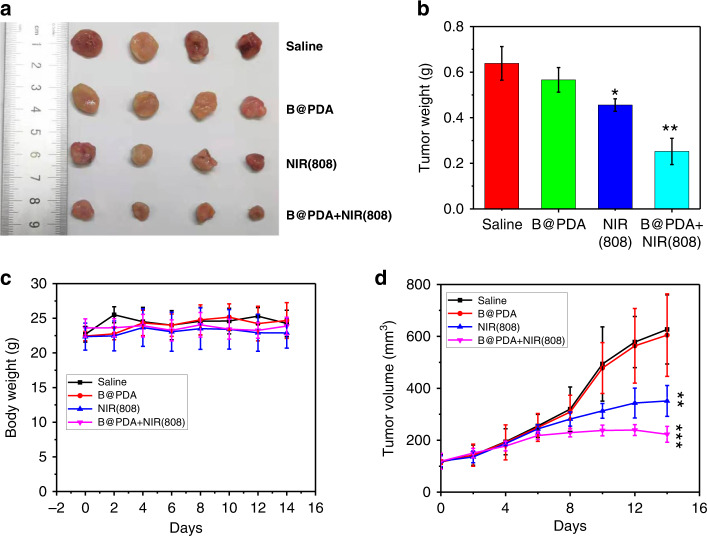
In vivo photothermal therapy efficacy of B@PDA platform. **a** Digital images of representative HCT-116 tumors after treatment. **b** Tumor weight comparison after treatment for four groups. **c** Tumor volume change during treatment (*n* = 4). **d** Mouse weight change during treatment. Error bar and mean value are defined as s.d. and mean, respectively. *P*-value was calculated by two-tailed Student’s *t* test (***P < 0.005, *P < 0.05)

Based on the excellent in vivo biocompatibility of the B@PDA platform, the in vivo antitumor study was carried out^90^. The mice with HCT-116 tumor were separated into four groups with different treatments: Group 1: PBS (control), Group2: B@PDA, Group 3: NIR (808 nm) and Group 4: B@PDA + NIR (808 nm). The intravenously injected dose of B@PDA was 5 mg kg^−1^ in related groups. Groups 3 and 4 were irradiated with an 808 nm laser (1 W cm^−2^, 10 min). After these treatments, the width and length of tumor were measured every 2 days and then calculated (Fig. 10a). Digital photos and weighs of representative tumors were also taken (Fig. 10b, c). Compared with the Control group, all Groups 2–4 exhibit tumor growth inhibition. Notably, Group 4 showed a better inhibition effect of tumor growth, demonstrating the enhanced therapeutic effect. Moreover, it is observed that there are no obvious side effects in the treatment process through weight measurement (Fig. 10d).

## Discussion

For the first time, we found that a top-down etching method can be used to fabricate borophene from a boride precursor and demonstrated its light-induced tumor therapy and imaging function. Ultrathin and large borophene nanosheets are successfully exfoliated. Through decoration of borophene with PDA, an effective cellular targeting system in the tumor environment is obtained. Besides novel fabrication, dual-modal photoacoustic and fluorescence imaging can be achieved to prove the tumor aggregation ability and facilitation of the phototherapy.

The novel fabrication technique of borophene as introduced here can be expanded to many other borides (MB_2_, M = Ti, Cr, V, Mn, Nb, Zr, Hf, Mo, etc.)^91^. Many unexplored borophene structures can be obtained through this fabrication technique as candidates for further light-induced tumor therapy and diagnosis. Other light-induced effects can also be explored, like photochemical, photomechanical effects or photobiological effects. Besides the tumor related theranostics, light interacting with these novel materials can provide more possibilities, such as treatment of cutaneous disorders, surgeries in ophthalmology, and interventional therapy in internal organs through fiber-optic delivery.

## Materials and methods

### Fabrications

The bulk AlB_2_ powder was bought from Sigma-Aldrich company. In all, 1 g of bulk AlB_2_ was added into HCl (30 mg/ml), respectively. Then the dispersion was stirred at 50 °C for 12 h. After reaction, the mixture was involved in an ultrasonic treatment. Finally, the borophene can be obtained through a centrifugation range of 3000 to 10000 rpm. The resulting sediment was then washed in deionized water. For HF etching, the method is the same.

1 mg of prepared borophene was dispersed with 1 mL ethanol. Dopamine hydrochloride aqueous solution (10 μL, 150 mg mL^−1^) and NaOH aqueous solution (50 μL, 10 mg mL^−1^) were mixed. Then, the mixture was stirred in dark for 2.5 h. Finally, the dispersion was centrifuged at 12,000 rpm for 10 min and B@PDA can be obtained. The sediment was washed two times and kept at 4 °C.

### Characterizations

High-resolution transmission electron microscopy (HRTEM, Tecnai G2 F30) was used to characterize the lateral size and crystal lattice of borophene. Atomic force microscopy (AFM, Bruker, Dimension Fastscan) is used to measure the thickness of borophene. The HRTEM image was obtained with an acceleration voltage of 300 kV. AFM image was scanned in 512 pixels per line. The crystal structure of borophene was determined by X-ray diffraction (XRD) patterns using Bruker D8 system. The chemical composition was confirmed by the X-ray photoelectron spectra (XPS) using ESCALAB 250Xi equipment. UV-Vis-NIR absorbance spectrum was measured employing ultraviolet spectrophotometry (Cary 60, Agilent). An 808 nm semiconductor laser (LSR808H, Lasever Inc.) was used to irradiate the borophene for generating heat. To measure the increasing temperature, both a thermocouple and infrared thermal imaging camera (FLIR E-75) were applied.

### Cell lines and reagents

The human colorectal cancer cells (HCT-116), cervical cancer cells (HeLa), lung adenocarcinoma cell line (A549) and human breast cancer cells (MCF7) were bought from American Type Culture Collection (ATCC). The cells were incubated in RPMI-1640 or Dulbecco’s Modified Eagle Medium (DMEM) supplied with 10% foetal bovine serum (FBS) and antibiotics (100 IU/mL penicillin and 100 µg/mL streptomycin), which were bought from GIBCO. Cell Counting Kit-8 and 4′, 6-Diamidino-2-phenylindole (DAPI) were bought from MCE (Monmouth Junction, NJ, USA). Enhanced chemiluminescence (ECL) kit, Mitochondrial Staining kit and Lysosome Staining Kit were obtained from BD (USA). Protease inhibitor cocktail and RIPA Lysis Buffer were bought from Sigma-Aldrich (Merck KGaA), Bicinchoninic acid (BCA) assay protein kit from EMD Millipore, and PVDF membranes from Bio Basic, Inc., Markham, ON, Canada.

### Animals

Four-week-old female BALB/C nude mice aged 5–6 weeks were bought from the Guangdong Medical Experimental Animal Center (GDMLAC) and were used for in vivo imaging and therapy experiments. All the protocols for in vivo experiments were approved by the Animal Welfare and Research Ethics Committee at Shenzhen University (ID: 2017003).

## Supplementary information

supplemental material
