# Supplementary material for: Light-induced tumor theranostics based on chemical-exfoliated borophene
Source: Light Sci Appl. 2022 Nov 11;11:324. doi: 10.1038/s41377-022-00980-9 (PMC9652458; doi:10.1038/s41377-022-00980-9)
Supplement: Supplementary file 1 — supplemental material [file 41377_2022_980_MOESM1_ESM.pdf]

Supplementary Information for Light-induced Tumor Theranostics based on Chemical-exfoliated Borophene

Zhongjian Xie[a,b,1], *Yanhong Duo*[b,i,1], *Taojian Fan*[b,1], *Yao Zhu*[c,1], *Shuai Feng*[d], *Chuanbo Li*[d], *Honglian Guo*[d], *Yanqi Ge*[b], *Shakeel Ahmed*[b], *Weichun Huang*[e], *Huiling Liu*[f], *Ling Qi*[g], *Rui Guo*[f], *Defa Li*[h*], *Paras N. Prasad*[j,*] and *Han Zhang*[b,*]

Affiliations

[a] Institute of Pediatrics, Shenzhen Children's Hospital, Shenzhen, Guangdong, China

[b] Shenzhen Engineering Laboratory of phosphorene and Optoelectronics; International Collaborative Laboratory of 2D Materials for Optoelectronics Science and Technology of Ministry of Education, Shenzhen Institute of Translational Medicine, Department of Otolaryngology, Shenzhen Second People's Hospital, the First Affiliated Hospital, Institute of Microscale Optoelectronics, Shenzhen University, Shenzhen, 518060, China

[c] Shenzhen Medical Ultrasound Engineering Center, Department of Ultrasonography，Shenzhen People’s Hospital, Second Clinical Medical College of Jinan University, First Clinical Medical College of Southern University of Science and Technology, Shenzhen 518020, China

[d]Optoelectronics Research Center, School of Science, Minzu University of China, Beijing, 100081, PR China

[e] Nantong Key Lab of Intelligent and New Energy Materials, College of Chemistry and Chemical Engineering, Nantong University, Nantong, 226019 Jiangsu, China

[f] Key Laboratory of Biomaterials of Guangdong Higher Education Institutes, Guangdong Provincial Engineering and Technological Research Centre for Drug Carrier Development, Department of Biomedical Engineering, Jinan University,

Guangzhou 510632, China
g Department of Core Medical Laboratory, the Sixth Affiliated Hospital of Guangzhou Medical University, Qingyuan People's Hospital, B24 Yinquan South Road, Qingyuan, Guang Dong Province, China
[h] Department of Laboratory Medicine, Shenzhen Children’s Hospital, Shenzhen, Guangdong, China
[i] Department of Microbiology, Tumor and Cell Biology (MTC), Karolinska Institute, Stockholm, Sweden
[j]Institute for Lasers, Photonics, and Biophotonics and Department of Chemistry, University at Buffalo, State University of New York, Buffalo, USA
[*]To whom correspondence should be addressed.
Email: hzhang@szu.edu.cn; guorui@jnu.edu.cn; pnprasad@buffalo.edu
[1] Z. Xie, Y. Duo, T. Fan and Y. Zhu contributed equally to this work.

Materials and methods

Cell viability assay

Cultured HCT-116, HeLa, MCF7 and A549 cells were placed in 96-well sterile plastic plates at an amount of 1.8×10^3 cells per well. Counting Kit-8 (CCK-8) was used to detect the cell viability. After 48 h of growth, the cells were treated in a dose-dependent of B @PDA. Then, the cells were added CCK-8 reagent (10 µL) and incubated for 0.5 hour at 37°C before assay. Absorbance of each well at 450 nm was measured through a microplate reader (Labsystem). The experiment was repeated in triplicate and the cell viability curve was plotted.

Endocytosis

The HCT-116 cells were cultured with Cy5-B@PDA for 30 min or 3 h, and then the cells were fixed with methyl alcohol for 15 min, permeabilized for 10 min with 0.1% Triton X-100, and blocked by 3 % bovine serum albumin (BSA) at room temperature for 2 h. Then, the coloring process was conducted with Mitochondrial Staining kit and Lysosome Staining Kit, respectively. Lastly, the cells were stained using DAPI for 10 min under dark condition at room temperature. For each step, cells were cleaned three times using PBS. With nail polish mounting, the cells were visualized and imaged on an Olympus FV1000 confocal microscope.

Detection of ROS

The HCT-116 cells were seeded on 12 well plates for one day. Then, the cells were incubated in the medium containing 25 ppm of B@PDA for 4h. After that, the cells were irradiated with an 808 nm laser (1 W cm^{-2}, 10 min). Then, the medium was wiped off and the cells were stained by 2′,7′-dichlorofluorescein (DCFH-DA), which is the fluorescent probes for ROS. Automated live-cell multispectral image was obtained through a High Content Analysis (HCA) Reader (ArrayScan XTI, Thermo Fisher Scientific Inc.) and the fluorescence images were recorded with the excitation wavelength of 504 nm and emission wavelength of 529 nm for DCFH-DA on channel

3. Image was analyzed through HCS Studio™ 2.0 software (Thermo Fisher Scientific Inc.).

Fluorescence imaging

The IVIS Imaging equipment (PerkinElmer Inc., Waltham, MA, USA) with filter sets was employed for living imaging. The camera was set with the maximum gain, a luminescent exposure time of 10 s and a binning factor of 4. Scanning parameters are as below: field of view of 12.5 cm, excitation/emission wavelength of 500/600 nm, fluency rate of 2 mW cm^{-2}.

PA imaging

PA imaging was conducted by Vevo LAZR-2100 PAUS system (VisualSonics Inc., Toronto, Canada). A linear acoustic array transducer (24 mm width, 21 MHz) was configured to the LAZR system. Laser wavelength of 808 nm was used.

Statistical Analyses

All data was shown as the mean ± the standard deviation (SD). Statistical analysis was carried on with SPSS 10.0 (SPSS, Inc., Chicago, IL, USA). $p<0.05$ was regarded as statistically significant (n = 3). Analysis of variance was conducted for all groups.

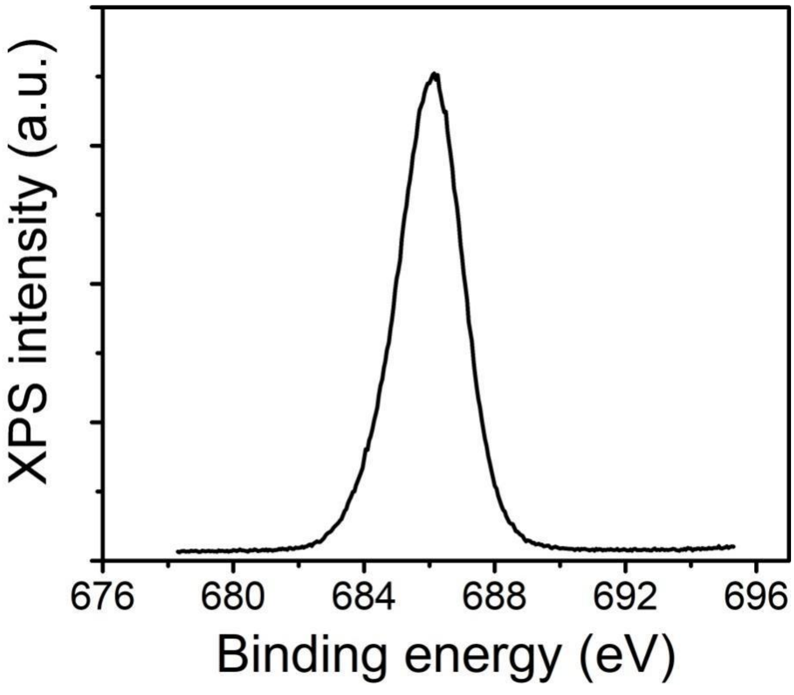

Figure S1. The XPS spectrum of F element for sample after HF etching of AlB_2.

Elements	Weight %	Atomic %
B	57.61	76.42
O	2.68	2.40
Al	39.38	20.93

Elements	Weight%	Atomic %
B	95.88	97.80
O	1.86	1.28
Al	2.26	0.92

Elements	Weight%	Atomic %
O	5.90	7.73
Al	35.19	27.32
F	58.9	64.95

Figure S2. EDS analysis of the bulk AlB2, the products after HCl and HF etching, respectively.

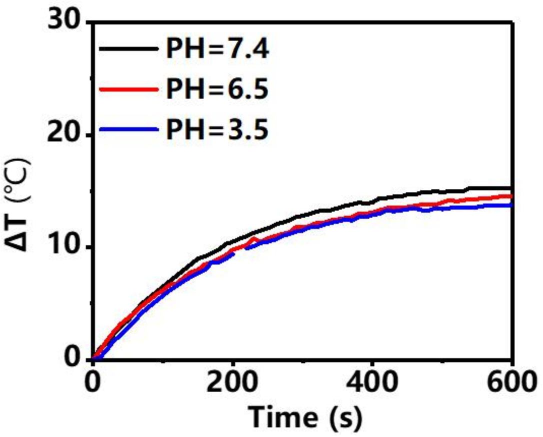

Figure S3 Photothermal performance of B@PDA (200 nm) under laser irradiation (808 nm, 1 W/cm^2) after being stored in aqueous solutions with different pH for 4 h. The results show that acidic conditions slightly affect the photothermal properties of B@PDA, which may be related to the accelerated degradation.

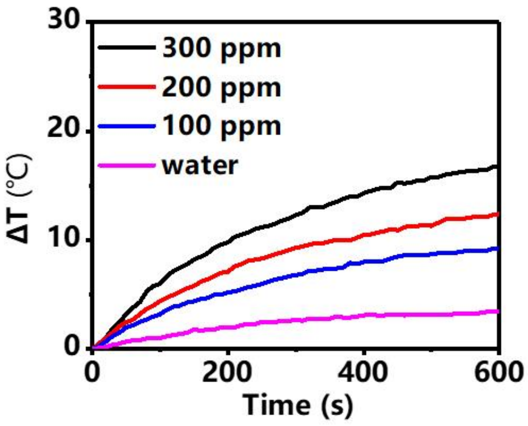

Figure S4 Photothermal performance of B@PDA with different concentration under NIR II laser irradiation (1064 nm, 1 W/cm^2, 10 min). Compared with water, the aqueous solution of B@PDA exhibited an obvious photothermal response in the NIR II region.

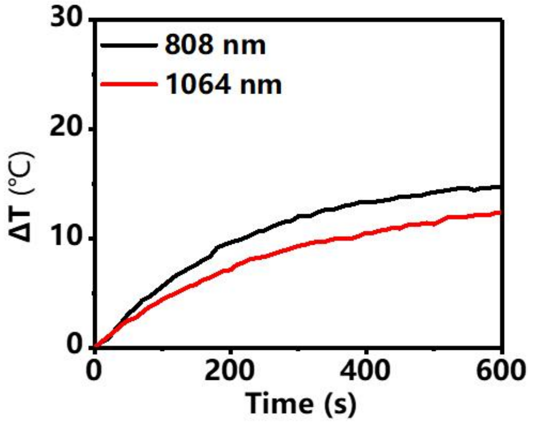

Figure S5 Photothermal performance of B@PDA (200 ppm) under irradiation with different wavelength (1064 nm or 808 nm, 1 W/cm^2, 10 min). The photothermal effect induced by the 1064 nm laser is weaker than that of the 808 nm laser.

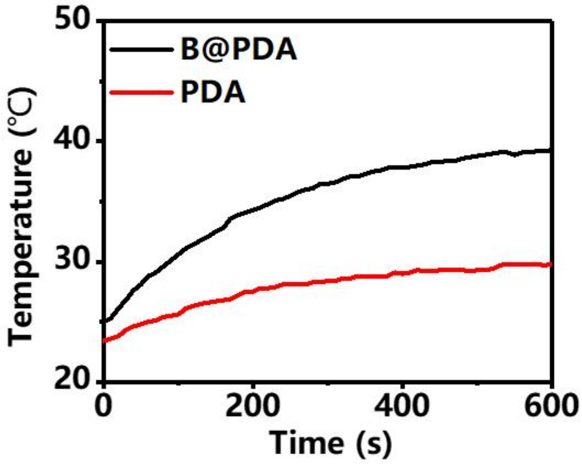

Figure S6 Photothermal performance of B@PDA (200 ppm) and pure PDA under irradiation (808 nm, 1 W/cm^2, 10 min). The preparation method of pure PDA is the same as that of B@PDA at the corresponding concentration. This data shows that the photothermal performance of B@PDA is significantly higher than that of pure PDA.

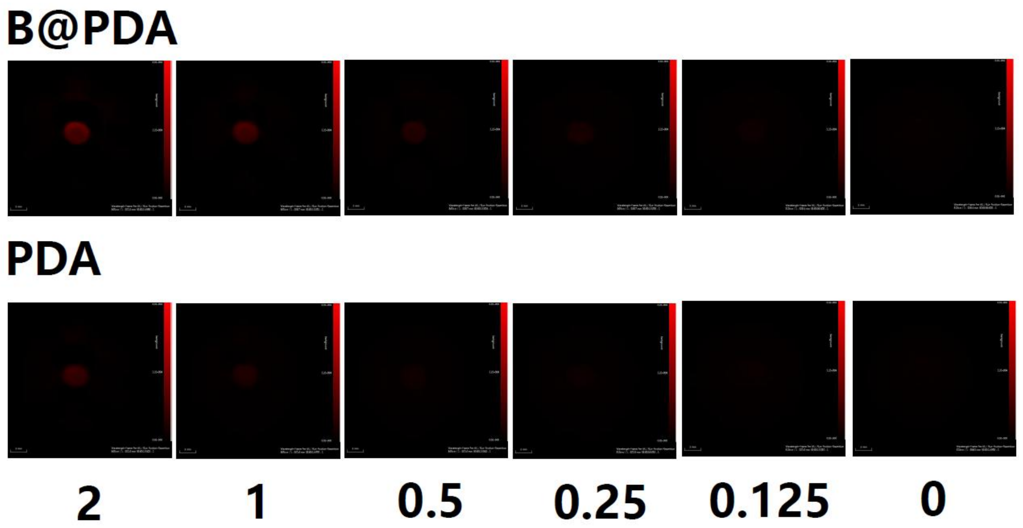

Figure S7 In vitro PA images of B@PDA and PDA as a function of concentration (0, 125, 250, 500, 1000, and 2000 μg mL^{-1}). This data shows that the photoacoustic signal of B@PDA is significantly higher than that of pure PDA. Data was conducted by MOST imaging system (inVision 128; iThera Medical, Germany).

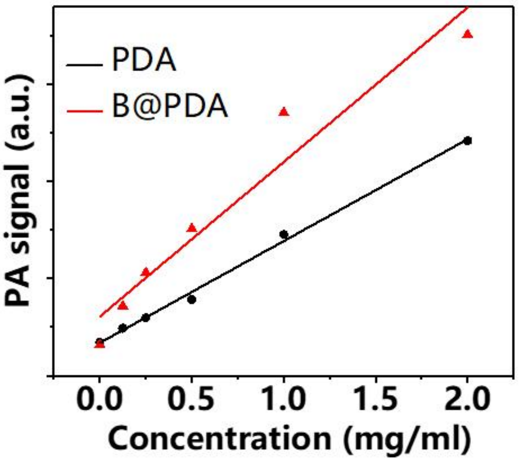

Figure S8 PA values of B@PDA and PDA as a function of concentration (0, 125, 250, 500, 1000, and 2000 μg mL^{-1}). Data was conducted by MOST imaging system (inVision 128; iThera Medical, Germany).

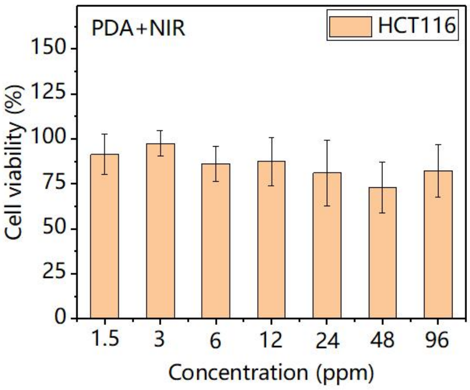

Figure S9 Relative viability of HCT-116 cell incubated with PDA for 4 h and after photothermal treatment (1 W cm^{-2}, 808 nm, 10 min). The preparation method of pure PDA is the same as that of B@PDA at the corresponding concentration. This data shows that the cell killing ability of B@PDA is significantly higher than that of pure PDA.

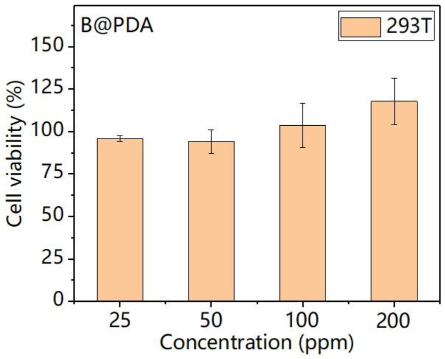

Figure S10 Relative viability of 293-T cell incubated with B@PDA for 48 h.

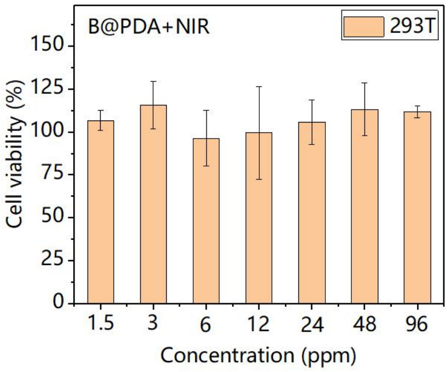

Figure S11 Relative viability of 293-T cell incubated with B@PDA for 4 h and after photothermal treatment (1 W cm^{-2}, 808 nm, 10 min).

Table S1. Extinction coefficient of several nanophotosensitizers

nanophotosensitizers	Extinction Coefficient	Wavelength (nm)	Reference

	($Lg^{-1}cm^{-1}$)		
Borophene	6.0	808	This work
Ge nanosheets	5.962	808	1
Graphene Oxide	3.6	808	2
AuNRs	3.9	808	3
Antimonene	5.58	808	4
Silicene	16	1064	5

Table S2. Photothermal conversion efficacy of several nanophotosensitizers

nanophotosensitizers	Photothermal conversion efficacy (%)	Wavelength (nm)	Reference
Borophene	32%	808	This work
Au nanorods	21%	808	6
$Cu_{2-x}Se$ nanocrystals	22%	808	7
MoS_2 nanosheets	23.8%	808	8
Graphene oxide (GO)	25%	532	9
Ti_3C_2 nanosheets	30.6%	808	10

References

[1] Feng C, Ouyang J, Tang Z, Kong N, Liu Y, Fu L et al. Germanene-Based Theranostic Materials for Surgical Adjuvant Treatment: Inhibiting Tumor Recurrence and Wound Infection. *Matter* 2020**;** **3**: 127-144.

[2] Robinson J, Tabakman S, Liang Y, Wang H, Sanchez C, Vinh, D et al. Ultrasmall Reduced Graphene Oxide with High Near-Infrared Absorbance for Photothermal Therapy. *J. Am. Chem. Soc.* 2011; **133**: 6825-6831.

[3] Xie H, Li Z, Sun Z, Shao J, Yu X, Guo Z et al. Metabolizable Ultrathin Bi_2Se_3 Nanosheets in Imaging-Guided Photothermal Therapy. *Small* 2016; **12**: 4136-4145

[4] Tao W, Ji X, Xu X, Islam M, Li Z, Chen S et al. Antimonene Quantum Dots: Synthesis and Application as Near-Infrared Photothermal Agents for Effective Cancer Therapy. *Angew. Chem. Int. Ed* 2017; **56**: 11896-11900.

[5] Lin H, Qiu W, Liu J, Yu L, Gao S, Yao H et al. Silicene: Wet-Chemical Exfoliation Synthesis and Biodegradable Tumor Nanomedicine. Adv. Mater. 2019; **31**: 1903013.

[6] Zeng J, Goldfeld D, Xia Y, A Plasmon-Assisted Optofluidic (PAOF) System for Measuring the Photothermal Conversion Efficiencies of Gold Nanostructures and Controlling an Electrical Switch. *Angew. Chem. Int. Ed* 2013; **52**: 4169-4173.

[7] Hessel C, Pattani V, Rasch M, Panthani M, Koo B, Tunnell J et al. Copper Selenide Nanocrystals for Photothermal Therapy. Nano Lett. 2011; **11**: 2560-2566.

[8] Liu T, Wang C, Gu X, Gong H, Cheng L, Shi X et al. Drug Delivery with PEGylated MoS2 Nano-sheets for Combined Photothermal and Chemotherapy of Cancer. Adv. Mater. 2014; **26**: 3433-3440.

[9] Meng D, Yang S, Guo L, Li G, Ge J, Huang Y et al. The enhanced photothermal effect of graphene/conjugated polymer composites: photoinduced energy transfer and

applications in photocontrolled switches. *Chem. Commun.* 2014; **50**: 14345-14348.
[10] Lin H, Wang X, Yu L, Chen Y, Shi J et al. Two-Dimensional Ultrathin MXene Ceramic Nanosheets for Photothermal Conversion. Nano Lett. 2017; **17**: 384-391.
